# Influence of shift work on periodontitis according to the occupation group

**DOI:** 10.1038/s41598-023-45222-z

**Published:** 2023-10-20

**Authors:** Seok-Ki Jung, Ho-Kyung Lim, Yujin Jeong, Sung Jae Lee, Jung Soo Park, In-Seok Song

**Affiliations:** 1 Department of Orthodontics, Korea University Guro Hospital, Seoul, Republic of Korea; 2 Department of Oral and Maxillofacial Surgery, Korea University Guro Hospital, Seoul, Republic of Korea; 3 Department of Biostatistics, Korea University College of Medicine, Seoul, Republic of Korea; 4 Department of Oral and Maxillofacial Surgery, Korea University Anam Hospital, 73 Goryeodae-ro, Seongbuk-gu, Seoul, 02841 Republic of Korea; 5 Department of Periodontology, Korea University Anam Hospital, 73 Goryeodae-ro, Seongbuk-gu, Seoul, 02841 Republic of Korea

**Keywords:** Dental diseases, Periodontitis

## Abstract

This study aimed to investigate the effects of shift work on periodontal disease in blue-and white-collar workers and to examine the interaction effects between occupation and work patterns. Data were collected from the Korea National Health and Nutrition Examination Survey conducted by the Korean Ministry of Health and Welfare for a total of nine years from 2007 to 2015. Participants with missing outcome variables were excluded from the analysis and a total of 32,336 participants were included in the final analysis. Univariable odds ratios (OR) were calculated using a logistic regression model with 95% confidence interval (CI). A multivariable logistic regression analysis was performed using the backward elimination method. The CONTRAST statement was used to analyze the interaction effect between occupation and work patterns. Multivariable logistic regression analysis revealed that interaction effects are present between the terms, occupational type and work pattern. Crude OR of shift work for periodontitis was 1.269 [CI 1.213–1.327, *P* < 0.05]. However, following adjustment for multiple confounding factors and the interaction effect term considered, this OR (1.269) increased to 1.381 [CI 1.253–1.523] in white-collar group while it decreased to 1.198 [1.119–1.283] in blue-collar. Crude OR of blue-collar (OR = 3.123, CI 2.972–3.281, *P* < 0.05) decreased to 1.151 [CI 1.049–1.262] when interaction effect to the shift work was considered. Shift work pattern increases the risk for periodontitis and this adverse effect is greater when white-collar workers are engaged comparing to blue-collar. The result of this study suggests that 24/7 lifestyle of the modern society poses health risks to the relevant people and the potential harm can be greater to white-collar workers.

## Introduction

### Irregular sleep pattern and systemic well-being

Quality sleep has become a popular topic in the modern era. Along with the development of technology and diversification in occupations, people no longer are devoted to traditional life cycle as in sleeping at night and working during daytime. While freed from standardized form of life, there are growing knowledge that discordance between 24 h day/night cycle and one’s sleep pattern affects adversely on various biological functions in human body that may even affect one’s life expectancy. Irregular or shifted sleep cycles are found to be associated with higher risk for type 2 diabetes, weight gain, coronary heart disease, stroke, and cancer^1^.

### Shift work and related sleep disorder

Shift work refers to a type of work being performed during outside of regular daytime hours, i.e., 09:00–18:00. Occupations engaging in 24-h restaurants, convenience stores, hospitals, and delivery work are the fields that are closely related to shift work. Shift workers may need alterations in their original sleep pattern in order to comply with shift work schedules and this leads to various systemic illnesses that are developed among shift workers^2^.

This biological harm can be primarily explained by circadian rhythmicity inherent in every organ and system of our body. When circadian disruption is occurred by atypical sleep pattern, metabolism and/or immunologic system cannot function properly, leading to aggravation of pre-existing systemic diseases like non-communicable chronic disease^3–5^. Secondly, it had been proven that people with irregular sleep schedules suffer from chronic sleep disturbances due to either shorter or longer but low-quality sleep, even showing a disorder called “shift work sleep disorder (SWSD)” in severe cases^6,7^. SWSD was found to be related to multiple diseases including type 2 diabetes, cardiovascular diseases, and various forms of cancers^1^.

### Periodontitis and shift work sleep disorder

Periodontitis is an inflammatory disease occurring in oral cavity which is receiving attentions from wide range of medical departments because strong mutual influences have been found between periodontitis and various non-communicable chronic diseases (NCDs)^8,9^. Given the demonstrated detrimental effect of shift work to systemic diseases and the relatedness between periodontitis and those diseases, it is a reasonable assumption that shift work and its related sleep disorder may also affect one’s periodontal status.

In this context, our group demonstrated in recent article that the risk of periodontitis is significantly increased among the subjects who are engaged in shift work^10^. It was our finding that irrespective of sleep duration, practicing occupational work during outside the daytime period (09:00–18:00), whether regular or irregular, increases odds ratio for periodontal disease among Korean adult population.

### Aim of the study

Therefore, our next question was which specific type of the occupation is mostly affected by shift work in terms of periodontal health. So called, white-collar and blue-collar jobs differ significantly in terms of the amount of physical demand, daily smoking, or dietary habits of the workers, and therefore, risk for various systemic diseases^11–13^. Blue-collar workers are more vulnerable to cancer, cardiovascular disease, and depressive disorders compared to other industries and job types^13^. On the other hand, white-collar workers are more prone to be exposed to psychosocial stresses at work showing higher prevalence for uncontrolled hypertension^11^. In term of periodontal health, blue-collar workers are found to have higher odds ratio for complete tooth loss in Japanese population^14^. Another population study in South Korea also revealed that blue-collar workers are more prone to have periodontal disease^15^. Since white-collar workers generally deal with psychologically-demanding tasks and large part of their work is done in front of the computer screen where blue-light is engaged, it was our hypothesis that SWSD may affect white-collar workers more strongly. The null hypothesis of this study was that shift work and the related sleep disorders does not affect periodontitis depending on the occupational type.

## Methods

### Survey participants

The data used in this study were derived from the Korea National Health and Nutrition Examination Survey, a nationwide cross-sectional survey conducted by the Korean Ministry of Health and Welfare between 2007 and 2015. The inclusion criteria were participants aged ≥ 19 years and those who answered the questionnaire regarding the longest occupation they had during their lifetime. Participants without records of work patterns or periodontal status were excluded from the study.

Of the 73,353 study participants, 56,039 were aged 19 years or older and 47,942 responded to the longest occupation survey column. Among them, 10,375 subjects without information on work patterns and 5231 subjects without periodontal records, were excluded. Therefore, data from 32,336 (44.08%) participants were used in the final statistical analysis. Among the total survey participants, the group with periodontal disease (Community periodontal index (CPI) ≥ 3) accounted for 41.72%, and the group without periodontal disease (CPI < 3) accounted for 58.28% of the total participants. This study was approved by the institutional review board (IRB) of the Korea University Anam Hospital. (IRB No. 2021AN0047; Seoul, Korea).

### Defining sociodemographic, lifestyle variables

Data on sex, age, and the number of missing teeth were recorded. The income level of the respondents was categorized by separating them into quartiles based on the average monthly income of their households. The educational level of the respondents was defined as the highest level of education they had completed. The participants were divided into two groups (smokers and non-smokers) based on their status of smoking at the time of the survey. A professional health researcher completed the questionnaires. Drinking status was classified as non-drinkers, light-to-moderate drinkers (1–30 g/day), and heavy drinkers (> 30 g/day)^16^. Information on sleep duration was collected through self-reported interviews and durations were categorized into three ranges: ≤ 5, 6–8, and ≥ 9 h/day.

### Defining metabolic health status

Hypertension (HTN) was defined as blood pressure (BP) ≥ 140/90 mmHg or medication of anti-hypertensive drug at the time of the survey. Prehypertension was defined as BP of 130–139/80–89 mmHg. Fasting blood glucose level of 126 mg/dL or higher was defined as impaired fasting glucose, and diabetes mellitus was defined as impaired fasting glucose and current use of antidiabetic medications. Presence of metabolic syndrome was determined if the participant has three or more of the following criteria fulfilled: (1) waist circumference ≥ 90 cm in males and ≥ 80 cm in females; (2) fasting triglycerides ≥ 150 mg/dl or use of lipid-lowering medication; (3) high-density lipoprotein cholesterol < 40 mg/dl in males and < 50 mg/dl in females or use of medication; (4) BP ≥ 130/85 mmHg or use of antihypertensive medication; and (5) fasting blood glucose ≥ 100 mg/dl or current use of anti-diabetic medication.

### Anthropometric and biochemical measurements

Following blood collection by trained staff members, samples were transported to the Central Testing Institute (NeoDin Medical Institute) for further analysis. White blood cell (WBC) counts were decided using laser flow cytometry (Sysmex XE-2100D). Serum cholesterol was measured enzymatically using chemical analyser (Hitachi 7600; Hitachi, Ltd.). BP was measured three times at 5-min intervals using a standard mercury sphygmomanometer (Baumanometer, WA Baum Co.) Body mass index (BMI) was determined through dividing body weight (kg) by squared height (m^2^).

### Occupational classification, work pattern, and periodontal disease evaluation

The survey on the longest occupation was performed by asking the following question: “What was the occupation you had for the longest duration in your lifetime?” Participants answered the questionnaire based on the nine major classification codes according to the Korean Standard Classification of Occupations and the occupations were mentioned as follows:

1. Managers
2. Professionals and associate professionals
3. Clerks
4. Service workers
5. Shop and market sales workers
6. Skilled agricultural and fishery workers
7. Craft and related trades workers
8. Plant and machine operators and assemblers
9. Elementary occupations

Managers, professionals, associate professionals, and clerks, who accounted for 62.99% of the total participants, were re-categorized as white-collar workers. The rest, including service workers, shop and market sales workers, skilled agricultural and fishery workers, craft and related trade workers, plant and machine operators and assemblers, and elementary occupations, were categorized as blue-collar workers.

Daytime work was defined as regular work between 6:00 and 18:00 on weekdays. Shift work included various work schedules, apart from regular daytime work and included evening work, night work, day/night shifts, 24-h shift work, split work, and irregular shift work. Respondents provided information on working hours and shift work based on the occupation they were engaged for the longest period in the lifetime. A total of 14,577 people (45.08%) were employed in daytime work and 17,759 (54.92%) in shift work.

Periodontitis was assessed using the CPI^17^. Periodontal probing depths (PPDs) were measured using CPI probe with a force of 20 g in 10 teeth (tooth numbers 11, 16, 17, 26, 27, 31, 36, 37, 46, and 47). CPI scores were determined based on following criteria: 0, healthy teeth; 1, gingival bleeding after probing; 2, calculus; 3, PPD of 4–5.5 mm; and 4, PPD ≥ 6 mm. Participants who had CPI ≥ 3 in their worst quadrant were considered to have periodontitis. Four public health dentists participated in the oral examinations, and the inter-examiner and inter-examiner reliabilities were higher than the k-coefficient of 0.85.

### Statistical analysis

Statistical analysis was performed using the Statistical Analysis System version 9.4. (SAS Institute Inc., Cary, NC, USA). According to the periodontal status of the participants, baseline characteristics were calculated using independent t-test and chi-square test. Data were presented as mean ± standard error and frequency (percentage) for continuous and categorical variables, respectively. Complete case analysis with covariate adjustment was used to handle missing data (Table S3). Statistical significance was set at α = 0.05.

### Univariable and multivariable logistic regression

Using univariable logistic regression model, unadjusted crude odd ratios (OR) were calculated for the variables that shows significant differences between CPI < 3 and CPI ≥ 3 groups. Codes were assigned to each group as 0 for CPI < 3, and 1 for CPI ≥ 3. Multivariable logistic regression analysis was performed using the backward elimination method. “Change-in-estimate” strategy was applied to identify putative confounders in the model. Variables that show significant increase of the ORs in the univariable model were considered as potential confounders (Table S4). Variables were removed from the model in order of level of significance (= *P*-value). Variable with the largest *P*-value was removed in consecutive order, and variables with the significance level > 0.25 were removed from the final model. Interaction effect refers to the role of a variable in an estimated model, and its effect on the dependent variable. When a variable has an interaction effect with some third variable, effects on the dependent variable differs depending on the level of the third variable. In this study, the CONTRAST statement was used to analyze the interaction effects between work patterns and occupation types. Using this interaction term, multiplicative effect of the two variables can be analyzed in multivariable logistic regression model.

### Ethics approval and consent to participate

This study was conducted in accordance with the guidelines of the Declaration of Helsinki and approved by the Institutional Review Board of Korea University Anam Hospital. (IRB No. 2021AN0047). The informed consent was waived by the Institutional Review Board of Korea University Anam Hospital.

## Results

### Baseline characteristics

Baseline characteristics of the study participants are presented in Table 1 and S1. The mean age of the participants in the periodontitis group was 57.7 ± 13.26 years and the non-periodontitis group was 41.1 ± 13.42 years (*P* < 0.05). The participants in the periodontitis group had more missing teeth than those in the non-periodontitis group, with an average of 7.9 ± 8.66 versus 1.1 ± 1.8, respectively (*P* < 0.05). Men (53.87%) were the more affected by periodontitis compared to female (*P* < 0.05). Variable including BMI, HbA1c, total cholesterol, WBC counts showed significant increases in periodontitis group (*P* < 0.05). Education, income, smoking, and drinking were also the variables showed significant differences (*P* < 0.05). Participants with HTN and diabetes occupied in higher percentage within periodontitis group (*P* < 0.05). In periodontitis group, blue-collar workers accounted for 77.57% and white-collar workers for 22.43% showing significant difference compared to the non-periodontitis group (*P* < 0.05). Work pattern (shift work/daytime work) also showed significant difference between two groups (*P* < 0.05).

**Table 1:** Baseline characteristics of this study according to the presence of periodontitis (N = 32,336).

	CPI < 3	CPI ≥ 3	*P*
	(*N* = 18,845)	(*N* = 13,491)	
	n (%)	n (%)	
Gender			< 0.0001
Male	8023 (42.57)	7267 (53.87)	
Female	10,822 (57.43)	6224 (46.13)	
Age, years (mean ± SD)	41.1 ± 13.42	57.7 ± 13.26	< 0.0001
Number of Missing Teeth	1.1 ± 1.8	7.9 ± 8.66	< 0.0001
Body mass index, kg/m	23.5 ± 3.42	24 ± 3.25	< 0.0001
HbA1c	5.7 ± 0.82	6.2 ± 1.2	< 0.0001
Total cholesterol	185.4 ± 34.59	192.1 ± 36.57	< 0.0001
WBC count	6.1 ± 1.66	6.4 ± 1.88	< 0.0001
Education			< 0.0001
Elementary school graduate or lower	2079 (11.04)	5582 (41.42)	
Middle school graduate	1498 (7.95)	1988 (14.75)	
High school graduate	7577 (40.22)	3582 (26.58)	
College graduate or higher	7683 (40.79)	2326 (17.26)	
Income			< 0.0001
Low	1853 (9.95)	3696 (27.92)	
Middle-low	4390 (23.57)	3595 (27.15)	
Middle-high	5879 (31.56)	3099 (23.41)	
High	6507 (34.93)	2850 (21.53)	
Smoking			< 0.0001
Nonsmoker	14,918 (79.32)	9909 (73.75)	
Smoker	3890 (20.68)	3527 (26.25)	
Drinking			< 0.0001
Nondrinker	8916 (47.31)	7267 (53.87)	
Light-to-moderate drinker	9356 (49.65)	5514 (40.87)	
Heavy drinker	573 (3.04)	710 (5.26)	
Hypertension			< 0.0001
Normal	10,752 (57.25)	4315 (32.14)	
Prehypertension	4509 (24.01)	3552 (26.46)	
Hypertension	3520 (18.74)	5558 (41.40)	
Diabetes			< 0.0001
Normal	14,012 (78.83)	7495 (59.87)	
Impaired fasting glucose levels	2907 (16.36)	3145 (25.12)	
Diabetes	855 (4.81)	1878 (15.01)	
Metabolic syndrome, no/yes	14,616 (77.56)/43 (0.23)	13,416 (99.45)/7511 (55.67)	< 0.0001
Cardiovascular disease, no/yes	18,674 (99.09)/171 (0.91)	13,045 (96.7)/445 (3.3)	< 0.0001
Chronic kidney disease, no/yes	18,801 (99.77)/43 (0.23)	13,416 (99.45)/74 (0.55)	< 0.0001
Occupation (re-categorized)			< 0.0001
Blue-collar	9903 (52.55)	10,465 (77.57)	
White-collar	8942 (47.45)	3026 (22.43)	
Work pattern (re-categorized)			< 0.0001
Shift work	9888 (52.47)	7871 (58.34)	
Daytime work	8957 (47.53)	5620 (41.66)	
Sleep duration/day, h			< 0.0001
≤ 5 h	2186 (11.60)	2491 (18.47)	
6–8 h	15,228 (80.82)	9808 (72.71)	
≥ 9 h	1428 (7.58)	1190 (8.82)	

SD, Standard deviation; CPI, community periodontal index.

### Univariable logistic regression model

In univariable logistic regression analysis, ORs were calculated to identify potential risk factors for periodontitis. Variables including age, number or missing teeth, BMI, HbA1c, total cholesterol, WBC count showed positive associations with periodontitis (*P* < 0.05, Table 2 and S2). Smoking and heavy drinking significantly increased OR for periodontitis to be 1.365 [1.296–1.438] and 1.52 [1.356–1.704], respectively. ORs increased in accordance with the severity of HTN and diabetes (ORs for HTN and diabetes, 3.934 [3.724–4.157] and 4.106 [3.769–4.473], respectively). In terms of occupation, blue-collar jobs showed significantly increased OR (3.123 [2.972–3.281], *P* < 0.05) for periodontitis compared to white-collar jobs. Shift work also increased OR significantly compared to daytime work (1.269 [1.213–1.327], *P* < 0.05). In terms of sleep duration, participants who slept ≤ 5 h/day (OR = 1.769 [1.662–1.884]) or ≥ 9 h/day (OR = 1.294 [1.193–1.403]) showed higher risk for periodontitis compared to the group with 6–8 h of sleep duration (*P* < 0.05).

**Table 2:** Univariable logistic regression analysis producing odds ratio of risk factors for periodontitis.

	Periodontal status		
	OR	95% CI	*P*
Age	1.089	1.087–1.091	< 0.0001
Number of missing teeth	1.435	1.420–1.451	< 0.0001
Body mass index, kg/m	1.052	1.045–1.059	< 0.0001
HbA1c	1.852	1.772–1.936	< 0.0001
Total cholesterol	1.005	1.005–1.006	< 0.0001
WBC count	1.108	1.093–1.122	< 0.0001
Gender			< 0.0001
Male		REF	
Female	0.635	0.607–0.664	
Education			< 0.0001
Elementary school graduate or lower		REF	
Middle school graduate	0.494	0.455–0.538	
High school graduate	0.176	0.165–0.188	
College graduate or higher	0.113	0.105–0.121	
Income			< 0.0001
Low		REF	
Middle-low	0.411	0.382–0.441	
Middle-high	0.264	0.246–0.284	
High	0.220	0.205–0.236	
Smoking			< 0.0001
Nonsmoker		REF	
Smoker	1.365	1.296–1.438	
Drinking			< 0.0001
Nondrinker		REF	
Light-to-moderate drinker	0.723	0.691–0.757	< 0.0001
Heavy drinker	1.52	1.356–1.704	< 0.0001
Hypertension			< 0.0001
Normal		REF	
Prehypertension	1.963	1.855–2.077	
Hypertension	3.934	3.724–4.157	
Diabetes			< 0.0001
Normal		REF	
Impaired fasting glucose levels	2.023	1.909–2.143	
Diabetes	4.106	3.769–4.473	
Metabolic syndrome			< 0.0001
No		REF	
Yes	2.752	2.622–2.887	
Cardiovascular disease			< 0.0001
No		REF	
Yes	3.724	3.118–4.449	
Chronic kidney disease			< 0.0001
No		REF	
Yes	2.412	1.655–3.514	
Occupation			< 0.0001
White-collar		REF	
Blue-collar	3.123	2.972–3.281	
Work pattern			< 0.0001
Daytime work		REF	
Shift work	1.269	1.213–1.327	
Sleep duration/day, h			< 0.0001
≤ 5 h	1.769	1.662–1.884	
6–8 h		REF	< 0.0001
≥ 9 h	1.294	1.193–1.403	< 0.0001

CI, Confidence interval; OR, odds ratio; REF, reference.

### Multivariable logistic regression model

In multivariable logistic regression model, variables including age, BMI, WBC count, gender, education, income, smoking, drinking, and diabetes maintained their significance. For the terms work pattern and occupation, interaction effects were calculated using the CONTRAST statement. When comparison is made between unadjusted ORs (Table 2) and interactions considered adjusted ORs (Table 3), it is noticeable that following adjustment, OR of shift work for periodontitis decreases in blue-collar group (from 1.269 to 1.198 [1.119–1.283]), whereas it increases in white collar (from 1.269 to 1.381 [1.253–1.523]). This implies that white-collar workers are affected by shift work to a greater extent compared to blue-collar workers in terms of risk for periodontal disease. Sleep duration was no longer a significant variable in our final model.

**Table 3:** Multivariable logistic regression analysis producing odds ratio of risk factors for periodontitis.

	Periodontal status		
	OR	95% CI	*P*
Age, years	1.081	1.078–1.084	< 0.0001
Body mass index, kg/m	1.011	1.002–1.02	0.0167
WBC count	1.086	1.067–1.105	< 0.0001
Gender			
Male		REF	
Female	0.711	0.663–0.762	< 0.0001
Education			< 0.0001
Elementary school graduate or lower		REF	
Middle school graduate	0.933	0.845–1.031	0.173
High school graduate	0.833	0.758–0.916	< 0.0001
College graduate or higher	0.713	0.635–0.801	< 0.0001
Income			< 0.0001
Low		REF	
Middle-low	0.916	0.835–1.004	0.0621
Middle-high	0.850	0.773–0.933	0.0007
High	0.776	0.704–0.854	< 0.0001
Smoking			
Nonsmoker		REF	
Smoker	1.742	1.613–1.881	< 0.0001
Drinking			0.0011
Nondrinker		REF	
Light-to-moderate drinker	0.979	0.919	0.5251
Heavy drinker	1.21	1.047	0.0096
Diabetes			< 0.0001
Normal		REF	
Impaired fasting glucose levels	1.131	1.055–1.213	0.0006
Diabetes	1.476	1.333–1.633	< 0.0001
Work pattern			
Daytime work		REF	
Shift work	1.381	1.253–1.523	< .0001
Occupation			
White collar		REF	
Blue collar	1.327	1.202–1.464	< .0001
Work pattern * Occupation			0.019
Shift work in Blue-collar (Ref. Daytime work)	1.198	1.119–1.283	< 0.0001
Shift work in White-collar (Ref. Daytime work)	1.381	1.253–1.523	< 0.0001
Blue-collar in Shift work (Ref. White collar)	1.151	1.049–1.262	0.003
Blue-collar in Daytime work (Ref. White collar)	1.327	1.202–1.464	< 0.0001

CI, Confidence interval; OR, odds ratio; REF, reference.

## Discussion

This study showed that effect of shift work on periodontal status differ according to the occupational types. White-collar workers are more likely to be affected by shift work compared to blue-collar workers in terms of prevalence of periodontitis (OR = 1.381 vs. 1.198, Table 3). This is the result drawn following adjustment for multiple potential confounding factors of periodontal disease including age, gender, BMI, WBC count, education and income level, smoking, drinking, and diabetes^18^.

This result is somewhat contrary to general understanding regarding health conditions of blue- and white-collar workers. According to the previous report, white-collar workers have lower risk for common chronic diseases and lower mortality rate for cardiovascular disease compared to blue-collar workers^19^. Also, in another publication where various risk indicators for chronic disease such as amount of exercise, physical work situation, smoking, diet, cardiorespiratory fitness, BMI, and blood pressure had been considered, blue-collar workers showed to have significantly higher clustering of those risk indicators (OR = 1.80 [1.71–1.90]) compared to white-collar workers^20^. Periodontitis is one of the non-communicable chronic diseases (NCDs) and proved to have close relationship with other NCDs through its contribution to low-grade systemic inflammation^21^. This explains the result of our univariable model revealing increased OR for periodontitis in blue-collar group (OR = 3.123).

The interesting part was to find interaction effect between occupational type and work pattern. Adverse effect of shift work on the periodontal status was greater in white-collar group when comparing to that in blue-collar group (Tables 2 and 3). Crude ORs for blue-collar and shift work group are 3.123 and 1.269, respectively. These numbers change after the interaction term is considered in the model. OR for blue-collar decreases from 3.123 to 1.151 when shift work is engaged, whereas OR for shift work increases in white-collar group from 1.269 to 1.381.

Shift work refers to work schedules apart from regular daytime period and are increasing in numbers as the modern society transforms continuously in accordance with the present needs. Especially, COVID-19 pandemic encouraged the emergence of various telecommuting jobs and invigoration of 24 h delivery industry. Shift work is found to be detrimental to overall health because it inevitably changes one’s wake/sleep pattern causing deterioration in sleep quality and disruption of the circadian rhythm^2,6,22^.

Alterations in sleep pattern in a manner which does not correspond to environmental day/night cycle, brings about disruption in one’s circadian rhythm. Every living organism has endogenous clock in biological systems such as endocrine, immune, and cardiovascular systems as well as renal and cerebral activities^23^. This clock is restrained by the environment cues like light, feeding, and body temperature. When misalignment is occurred between external and internal circadian clock due to atypical sleep pattern, various physiological activities governed by circadian rhythm cannot function properly. In periodontal aspect, gingival fibroblast, which in charge of first barrier defense at the bacterial front, is found to be regulated by the clock gene CLOCK/BMAL1^24^. Furthermore, immune cells including neutrophils and macrophages express strong rhythmicity, thereby showing clock-controlled manner in their immunological function^25^.

White-collar jobs include managers, professionals, associate professionals, and clerks. These workers tend to spend most of their working hours in front of a computer screen, overly exposed to blue light. Since blue-light itself creates a significant disturbance in the circadian rhythm^26–28^, combination of shift work and blue-light can be even more harmful. One of the white-collar jobs well known for shift work is the hospital nurses. Their work is strictly engaged to the shift schedules and weakened immunity and poor physical conditions along with such working condition had been reported^29^. However, the exact mechanism of why white-collar workers are more vulnerable to shift work is still ambiguous and a matter for further research.

Nevertheless, the strength of our result is that this is the first study to consider interaction effect between occupational type and shift working pattern. Given the close relationship between periodontal disease and systemic well-being, our result may offer a new perspective to future research regarding shift work and other chronic diseases.

The limitations of this study include its cross-sectional nature, possible underestimation of participants’ periodontal status due to the CPI system, and use of self-recorded questionnaires. In addition, several significant data are missing due to the nature of the national surveillance data. Factors like sleep quality, exact duration of the occupational engagement, and oral hygiene status may strengthen the weight of the result. Therefore, further longitudinal study with more detailed data is required in order to confirm the correlation between periodontal disease and the effect of occupational shift work. Also in the future research, categorization of the occupations could be in a different manner, such as white-, blue-, and pink-collar (service and sales workers), for more concrete result. Within the limitations of this study, the effect of shift work seems particularly noticeable in white-collar workers compared to blue-collar workers. The result of this study suggests that 24/7 lifestyle of the modern society poses health risks to the relevant people and the potential harm can be greater to white-collar workers. To determine the range of influence in detail, further investigations with more sophisticated data collection are necessary.

In conclusion, adverse effect of shift work on periodontal disease is greater in white-collar group compared to blue-collar workers.

### Supplementary Information

Supplementary Tables.

## Data Availability

The datasets used and/or analyzed during the current study are available from the corresponding author upon request.
