# Supplementary material for: Influence of shift work on periodontitis according to the occupation group
Source: Sci Rep. 2023 Oct 20;13:17921. doi: 10.1038/s41598-023-45222-z (PMC10589210; doi:10.1038/s41598-023-45222-z)
Supplement: Supplementary file 1 — Supplementary Tables. [file 41598_2023_45222_MOESM1_ESM.docx]

**Supplement**

**Table S1. Prevalence of periodontitis according to Occupation and work pattern**

|  | CPI < 3  (*N* = 18845)  n (%) | CPI ≥ 3  (*N* = 13491)  n (%) | *P* |
| --- | --- | --- | --- |
| Occupation  Managers  Professionals and associate professionals  Clerks  Service workers  Shop & market sales workers  Skilled agricultural and fishery workers  Craft and related trades workers  Plant and machine operators and assemblers  Elementary occupations | 459 (2.44)  4340 (23.03)  4143 (21.98)  1885 (10.00)  2507 (13.30)  1034 (5.49)  1566 (8.31)  1252 (6.64)  1659 (8.80) | 391 (2.90)  1186 (8.79)  1449 (10.74)  1009 (7.48)  1615 (11.97)  3129 (23.19)  1607 (11.91)  1248 (9.25)  1857 (13.76) | <0.0001 |
| Work pattern  Daytime  Evening shift (14:00~24:00)  Night shift (21:00~08:00)  Day/night regular rotating shift  24 h rotating shift  Split shift (more than two per day)  Irregular rotating shift  The others | 8957 (47.53)  1379 (7.32)  373 (1.98)  377 (2.00)  129 (0.68)  145 (0.77)  205 (1.09)  7280 (38.63) | 5620 (41.66)  452 (3.35)  201 (1.49)  169 (1.25)  195 (1.45)  79 (0.59)  116 (0.86)  6659 (49.36) | <0.0001 |

**Table S2. Univariate logistic regression analysis for occupational type**

|  | CPI < 3  (*N* = 18845)  n (%) | CPI ≥ 3  (*N* = 13491)  n (%) | *P* |
| --- | --- | --- | --- |
| Occupation  Managers  Professionals and associate professionals  Clerks  Service workers  Shop & market sales workers  Skilled agricultural and fishery workers  Craft and related trades workers  Plant and machine operators and assemblers  Elementary occupations | 0.321  0.411  0.628  0.756  3.552  1.205  1.17  1.314 | REF  0.276-0.372  0.354-0.476  0.538-0.734  0.652-0.877  3.051-4.136  1.035-1.402  1.001-1.368  1.131-1.527 | <0.0001  <0.0001  <0.0001  <0.0001  0.0002  <0.0001  0.0162  0.0484  0.0004 |

**Table S3. Missing data**

Amount of missing data in the study population (n=**32336**) for each variable considered. Missing data were handled with complete case analyses with covariates adjustment (Groenwold et al. 2012).

|  | **Collected data, N (%)** | **Missing data, N(%)** |
| --- | --- | --- |
| Periodontitis (outcome variable) | 32336 (100.0) | 0 (0.0) |
| Occupation | 32336 (100.0) | 0 (0.0) |
| Work pattern | 32336 (100.0) | 0 (0.0) |
| Age | 32336 (100.0) | 0 (0.0) |
| Body mass index | 32271 (99.80) | 65 (0.20) |
| Gender | 32336 (100.0) | 0 (0.0) |
| Educational Level | 32315 (99.94) | 21 (0.06) |
| Income | 31869 (98.56) | 467 (1.44) |
| Smoking Status | 32244 (99.72) | 92 (0.28) |
| Drinking | 32336 (100.0) | 0 (0.0) |
| Diabetes Status | 30292 (93.68) | 2044 (6.32) |
| WBC Count | 31037 (95.98) | 1299(4.02) |

**Table S4. Confounders selection for CPI case definition**

Below are the Change-in-estimate (OR) according to the work pattern caused by each putative confounder for the prevalence of periodontitis (CPI). Each putative confounder was tested separately. When the estimate change of the OR after adjustment exceeds 10% compared to the crude OR, the potential confounder was considered as the putative confounder.

| **CPI confounders selection** | | |
| --- | --- | --- |
| OR (95% CI) crude = 1.269 (1.213 - 1.327) | | |
| 10% 0.127 | | |
| Change if OR < 1.142, or  OR > 1.396 | | |
|  | | |
| **POTENTIAL CONFOUNDER** | **OR (work pattern)** | **95% CI (work pattern)** |
| Occupation | 1.206 | (1.152-1.263) |
| Age | 1.264 | (1.199-1.332) |
| Gender | 1.306 | (1.248-1.366) |
| Educational Level(confounder) | 1.108 | (1.055-1.163) |
| Income | 1.172 | (1.119-1.228) |
| Smoking Status | 1.275 | (1.219-1.333) |
| Drinking | 1.256 | (1.201-1.314) |
| Diabetes Status | 1.310 | (1.249-1.374) |
| Hypertension | 1.286 | (1.227-1.347) |
| WBC Count | 1.276 | (1.22-1.335) |
| Body mass index | 1.265 | (1.208-1.324) |

Abbreviations: OR, odds ratio; CI, confidence interval.

**None of the putative confounders except education level caused a change-in-estimate greater of 10%.**
